# Remarkable pathological response to neoadjuvant tepotinib in lung adenocarcinoma with *MET* exon 14 skipping mutation: A case report

**DOI:** 10.1111/1759-7714.15459

**Published:** 2024-09-29

**Authors:** Rongzhen Li, Xiaoyan Liu, Yan Xu, Jing Zhao, Wei Zhong, Xiaoxing Gao, Minjiang Chen, Mengzhao Wang

**Affiliations:** ^1^ Department of Respiratory and Critical Care Medicine Peking Union Medical College Hospital, Chinese Academy of Medical Sciences and Peking Union Medical College Beijing China; ^2^ Chinese Academy of Medical Sciences and Peking Union Medical College Beijing China

**Keywords:** *MET* exon 14 skipping mutation, neoadjuvant, non‐small cell lung cancer, tepotinib

## Abstract

Mesenchymal–epithelial transition (*MET*) exon 14 (*MET*ex14) skipping mutation is a rare (3%–4%) driver mutation in non‐small cell lung cancer (NSCLC). Tepotinib, a selective MET inhibitor, has shown promise in treating *MET*ex14 skipping‐mutated NSCLC. However, its feasibility for perioperative application remains unclear. This report describes a 60‐year‐old man with stage IIIA (cT2N2M0) lung adenocarcinoma harboring a *MET*ex14 skipping mutation. After initial treatment with savolitinib was discontinued due to grade 4 transaminitis, the patient was switched to tepotinib, resulting in significant tumor regression. Six months later, further shrinkage was observed, and surgery revealed remarkable pathological response with no residual tumor in lymph nodes (ypT2N0M0, IB). Postoperative tepotinib continued, with no relapse at 6‐month follow‐up. This case highlights the potential of tepotinib as neoadjuvant therapy for resectable *MET*ex14 skipping‐mutated NSCLC, warranting further clinical trials.

## Graphical abstract

This report describes a 60‐year‐old man with stage IIIA (cT2N2M0) lung adenocarcinoma harboring a *MET*ex14 skipping mutation. After initial treatment with savolitinib was discontinued due to grade 4 transaminitis, the patient was switched to tepotinib. Six months later, significant tumor shrinkage was observed, and surgery revealed remarkable pathological response with no residual tumor in lymph nodes (ypT2N0M0, IB). Postoperative tepotinib continued, with no relapse at 6‐month follow‐up. The arrows in different colors highlight different involved sites: yellow arrows — primary tumor lesion; red arrows — 7th lymph node; blue arrows — right hilar lymph node.

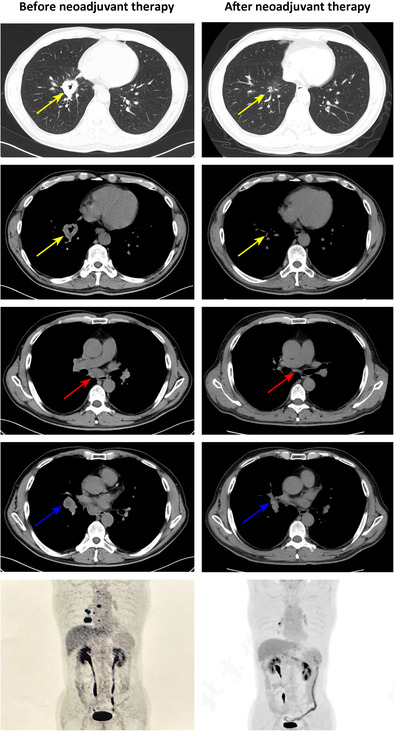

## INTRODUCTION

The mesenchymal–epithelial transition (MET) factor, encoded by the *MET* gene, plays a crucial role in the cancer progression of non‐small cell lung cancer (NSCLC). *MET* exon 14 (*MET*ex14) skipping mutations are primary oncogenic drivers occurring in approximately 3%–4% of NSCLC patients.^1^ These mutations are associated with distinct clinicopathological characteristics, including an older age of onset, a higher frequency in sarcomatoid and adenosquamous carcinoma compared to adenocarcinoma.^1^, ^2^ Furthermore, in the era of chemotherapy and immunotherapy, NSCLC patients with *MET*ex14 mutations often have a worse prognosis than those without the mutations.^3^, ^4^

Recently, targeted therapy for *MET*ex14 skipping mutation has shown promising progress in treating advanced NSCLC. Studies have demonstrated significant clinical benefits, including improved overall survival and progression‐free survival (PFS) rates, as well as notable responses to selective MET inhibitors such as savolitinib, capmatinib, and tepotinib.^5^, ^6^, ^7^ However, the role of MET inhibitors in perioperative therapy for resectable NSCLC remains unclear.

Tepotinib, approved in December 2023 in China, is used for the treatment of advanced NSCLC with *MET*ex14 skipping variants. Herein, we report the first case of resectable *MET*ex14‐positive NSCLC receiving tepotinib as neoadjuvant therapy followed by surgical resection.

## CASE REPORT

**FIGURE 1:**
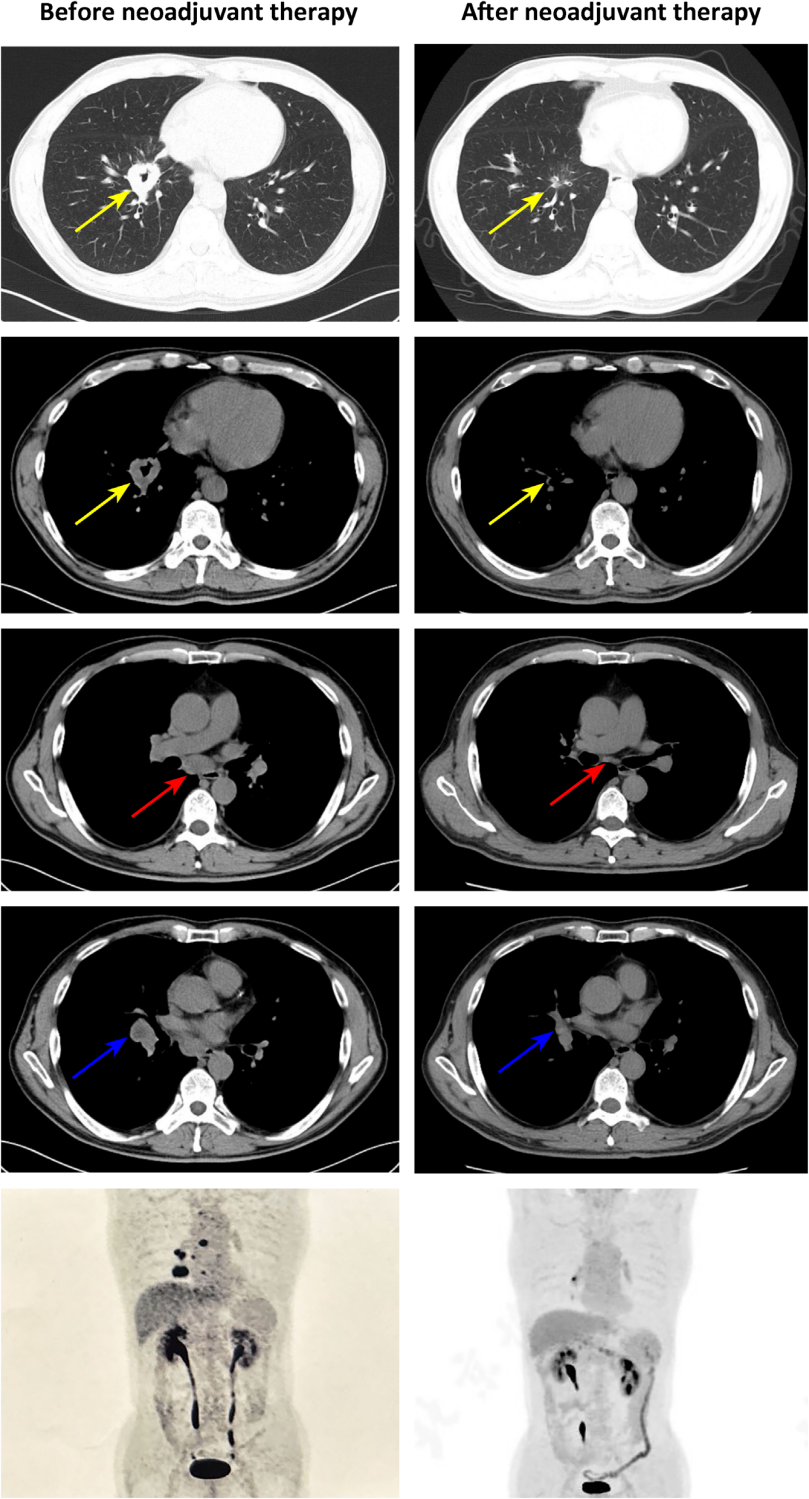
Imaging examination before and after neoadjuvant treatment. Chest computed tomography (CT) scan and positron emission tomography/CT (PET/CT) images of the right lung adenocarcinoma (yellow arrows), N7 (red arrows), and right hilar lymph (blue arrows) nodes before and after neoadjuvant treatment.

**FIGURE 2:**
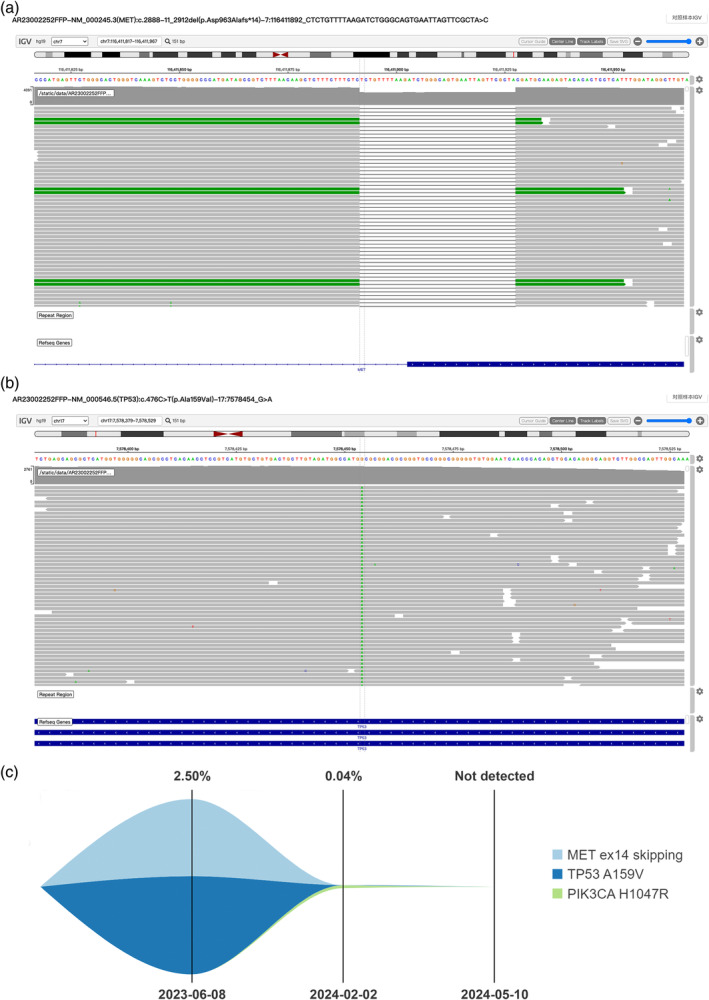
Genetic alterations identified in tumor tissue and peripheral blood. (a, b) Next‐generation sequencing (68‐gene panel, Lung Core, Burning Rock Biotech) using tumor issue revealed a *MET*ex14 skipping and a point mutation in *TP53*. (c) Dynamic monitor of circulating tumor DNA (ChosenLung/ChosenPace, ChosenMed) during treatment.

A 60‐year‐old man with a 30‐year smoking history was admitted to Peking Union Medical College Hospital (PUMCH) on May 29, 2023, due to multiple ground glass pulmonary nodules for 5 years and a new present right lower lobe solid nodule discovered by chest scan without obvious symptoms. Computed tomography (CT) scan and positron emission tomography/CT (PET/CT) revealed a 3.4 × 2.8‐cm soft tissue mass (SUVmax 9.2) in the basal segment of the lower lobe of the right lung, with enlarged mediastinal (seventh) and right hilar lymph nodes (SUVmax 10.3; Figure 1). Magnetic resonance imaging of the brain showed no evidence of central nervous system involvement. An endobronchial ultrasound‐guided transbronchial needle aspiration of mediastinal lymph nodes in station N7 established the diagnosis of a metastatic lung adenocarcinoma, with immunohistochemical manifestation as CK7(+), P40(−), TTF‐1(+), Ki‐67 (index 80%), and P53(+). Next‐generation sequencing (68‐gene panel, Lung Core, Burning Rock Biotech) using tumor tissue identified a *MET*ex14 skipping mutation (abundance: 23.55%), a point mutation in *TP53* and no other driver mutations (Figure 2a,b).

The patient's disease was evaluated as cT2N2M0, stage IIIA (UICC eighth edition). A multidisciplinary team (MDT) considered it a resectable case with single‐station N2 involvement. Given the significant progress in MET inhibitors for patients with advanced NSCLC harboring *MET*ex14 skipping mutations, the team discussed the options of curative chemoradiotherapy and induction therapy with the patient. The patient declined chemoradiotherapy and preferred neoadjuvant therapy followed by surgery. After being adequately informed about the potential benefits and risks of the standard chemoimmunotherapy regimen and non‐standard target treatment option, the patient ultimately chose neoadjuvant targeted therapy.

On June 14, 2023, after obtaining informed consent from the patient and receiving off‐label use approval from Pharmacy and Therapeutics Committee of PUMCH, savolitinib 600 mg once daily was prescribed and was discontinued due to grade 4 transaminitis on July 21, 2023. Following the patient's recovery, tepotinib was administered orally at 450 mg once daily starting on August 21, 2023. The follow‐up CT scan and PET/CT revealed a significant reduction in the mass, now a 1.0 × 0.9‐cm nodule (SUVmax 0.9). An elevated uptake of 18F‐FDG (SUVmax 7.6) was observed in a small right hilar lymph node, without any suspected metastasis in the mediastinal lymph nodes (Figure 1). The main adverse effects during tepotinib therapy were diarrhea, fatigue, and edema. All of the adverse events were grade 1. Following MDT evaluation, the patient underwent video‐assisted thoracic surgery right lower lobectomy plus lymph node dissection on January 4, 2024. The only surgical complication was asymptomatic hypersensitive cardiac troponin I elevation. Postoperative pathological examination confirmed invasive adenocarcinoma involving the main bronchus, with all 25 dissected lymph nodes being free of tumor cells, resulting in a pathological stage of ypT2N0M0, IB. After surgery, the patient continued adjuvant targeted therapy with tepotinib. By 1 and 4 months after surgery, circulating tumor DNA analysis did not detect *MET*ex14 skipping mutation (Figure 2c). No disease relapse was observed at the 6‐month postoperative follow‐up.

## DISCUSSION

To our best knowledge, this is the first case of *MET*ex14 skipping‐mutated lung adenocarcinoma receiving tepotinib as neoadjuvant therapy followed by surgery. In this case, neoadjuvant tepotinib demonstrated favorable efficacy with a tolerable safety profile.

Based on clinical trial data, neoadjuvant chemoimmunotherapy is recently recommended for resectable NSCLC. However, some oncogenic drivers, such as sensitizing epidermal growth factor receptor (*EGFR*) mutation and anaplastic lymphoma kinase (*ALK*) rearrangements, have shown less benefit from immunotherapy for advanced NSCLC. It remains unclear whether targetable driver mutations reduce the efficacy of neoadjuvant chemoimmunotherapy, as existing studies have produced controversial findings. The KEYNOTE‐671 trial (NCT03425643) reported a significant event‐free survival (EFS) benefit with neoadjuvant immunotherapy (hazard ratio [HR] 0.09, 95% confidence interval [CI]: 0.01–0.74) for *EGFR*‐mutated patients (*n* = 33).^8^ However, in the LCMC3 trial (NCT02927301), no patient with known *EGFR* mutation (*n* = 7) achieved major pathological response.^9^ Furthermore, the subgroup analysis in AEGENT trial (NCT03800134) suggested no clear evidence of EFS benefit with immunotherapy (HR 0.86, 95% CI: 0.35–2.19) in *EGFR*‐mutated patients (*n* = 51).^10^

A recent study found that in patients with stage III unresectable *EGFR*‐mutated NSCLC, consolidation osimertinib was superior to durvalumab after concurrent chemoradiation.^11^ Moreover, some clinical trials have demonstrated the feasibility of neoadjuvant targeted therapy for *EGFR*‐positive NSCLC.^12^, ^13^ Therefore, targeted therapy may be a more appropriate neoadjuvant treatment option for patients with targetable driver mutations.

The evidence for the optimal regimen for resectable NSCLC with *MET*ex14 skipping mutations in the perioperative setting is limited. Previous studies on neoadjuvant chemoimmunotherapy did not perform survival analysis in the subgroup of patients harboring *MET*ex14 skipping variants. Only one retrospective study found that *MET*ex14 skipping might shorten the EFS after neoadjuvant chemoimmunotherapy.^14^ For advanced *MET*ex14 skipping‐mutated NSCLC, a real‐world study suggested that first‐line MET inhibitor had greater efficacy than immunomonotherapy or chemoimmunotherapy.^15^ However, only a few case reports have discussed the efficacy and safety of savolitinib in the neoadjuvant setting.^16^, ^17^, ^18^ In the present case, the patient had radiographic downstaging after neoadjuvant tepotinib, and surgery was successfully performed. A remarkable pathological response and postoperative nodal downstage (ypN0) were observed. Regarding safety, the patient initially treated with savolitinib but experienced severe transaminitis leading to discontinue savolitinib. After switching to tepotinib, neither transaminitis nor other serious adverse events were observed.

The patient also harbors a *TP53* missense mutation that leads to the inactivation of the TP53 protein. Mutations in the tumor suppressor *TP53* gene are associated with the development of lung cancer and contribute to poor prognosis and treatment resistance.^19^ In *MET*ex14 skipping‐mutated NSCLC, *TP53* alterations are the most common co‐mutations. The correlation between *TP53* co‐mutations and the efficacy of tepotinib remains unclear, although the clinical trial has suggested a trend toward reduced PFS in patients with mutated *TP53* treated with tepotinib.^6^

This study has several limitations. First, the use of tepotinib for neoadjuvant therapy is currently considered off‐label. While this patient benefited from the treatment, large‐scale prospective studies are needed to validate the feasibility of this therapeutic strategy. Additionally, due to the short follow‐up, the overall survival benefit remains unclear. Lastly, targeted therapies for other rare mutations in NSCLC lack first‐line indications, and clinical research data is limited.

In summary, tepotinib may be a promising therapeutic option for patients with resectable *MET*ex14 skipping‐mutated NSCLC, but further studies are requested to confirm its efficacy.

## AUTHOR CONTRIBUTIONS

**Rongzhen Li**: Investigation, data curation, visualization, writing – original draft. **Xiaoyan Liu:** Resources, data curation. **Yan Xu:** Resources, data curation. **Jing Zhao:** Resources, data curation. **Wei Zhong:** Resources, data curation. **Xiaoxing Gao:** Resources, data curation. **Minjiang Chen:** Conceptualization, investigation, resources, data curation, writing – review & editing, supervision, project administration. **Mengzhao Wang:** Resources, data curation, supervision, project administration, funding acquisition.

## FUNDING INFORMATION

This work was supported by the National High Level Hospital Clinical Research Funding (2022‐PUMCH‐B‐106 to MW).

## CONFLICT OF INTEREST STATEMENT

The authors declare no conflicts of interest.

## Data Availability

The original contributions presented in the study are included in the article. Further inquiries can be directed to the corresponding author.
